# Diversified Resistance Mechanisms in Multi-Resistant *Lolium* spp. in Three European Countries

**DOI:** 10.3389/fpls.2020.608845

**Published:** 2020-12-15

**Authors:** Laura Scarabel, Silvia Panozzo, Donato Loddo, Solvejg K. Mathiassen, Michael Kristensen, Per Kudsk, Thomas Gitsopoulos, Ilias Travlos, Eleni Tani, Dimosthenis Chachalis, Maurizio Sattin

**Affiliations:** ^1^Institute for Sustainable Plant Protection (IPSP-CNR), National Research Council of Italy, Padua, Italy; ^2^Department of Agroecology, Aarhus University, Flakkebjerg, Denmark; ^3^Institute of Plant Breeding and Genetic Resources, Hellenic Agricultural Organization-Demeter, Thessaloniki, Greece; ^4^Department of Crop Science, Agricultural University of Athens, Athens, Greece; ^5^Benaki Phytopathological Institute, Weed Science Laboratory, Athens, Greece

**Keywords:** ryegrass, target-site resistance, enhanced gene expression, metabolism, multiple herbicide resistance

## Abstract

Annual ryegrass species (*Lolium* spp.) infest cereal crops worldwide. Ryegrass populations with multiple resistance to the acetyl coenzyme A carboxylase (ACCase) and acetolactate synthase (ALS) inhibitors are an increasing problem in several European countries. We investigated the resistance pattern and level of resistance in ryegrass populations collected in Denmark, Greece and Italy and studied the diversity of mechanisms endowing resistance, both target-site and metabolism based. All populations showed high resistance indexes (RI) to the ALS inhibitors, iodosufuron-methyl-sodium + mesosulfuron-methyl (RI from 8 to 70), whereas the responses to the two ACCase inhibitors, clodinafop-propargyl and pinoxaden, differed. The Greek and Italian populations were moderately to highly resistant to clodinafop (RI > 8) and showed low to moderate resistance to pinoxaden (RI ranged from 3 to 13) except for one Italian population. In contrast, the Danish *Lolium* populations showed low to moderate resistance to clodinafop (RI ranged from 2 to 7) and only one population was resistant to pinoxaden. Different mutant *ACCase* alleles (Leu_1781_, Cys_2027_, Asn_2041_, Val_2041_, Gly_2078_, Arg_2088_, Ala_2096_) and *ALS* alleles (Gly_122_, Ala_197_, Gln_197_, Leu_197_, Ser_197_, Thr_197_, Val_205_, Asn_376_, Glu_376_, Leu_574_) endowing resistance were detected in the Greek and Italian populations. In several plants, no mutated *ALS* and *ACCase* alleles were found showing a great heterogeneity within and among the Greek and Italian populations. Conversely, no mutant *ACCase* alleles were identified in the four Danish populations and only one mutant *ALS* allele (Leu_574_) was detected in two Danish populations. The expression level of nitronate monooxygenase (*NMO*), glutathione *S*-transferase (*GST*) and cytochrome P450s (*CYP72A1* and *CYP72A2*) varied broadly among populations and individual plants within the populations. Constitutive up-regulation of *GST*, *CYP72A1* and *CYP72A2* was detected in resistant plants respect to susceptible plants in one Danish and one Italian population. It appears that the mechanisms underlying resistance are rather complex and diversified among *Lolium* spp. populations from the three countries, coevolution of both target-site resistance and metabolic based herbicide resistance appears to be a common feature in Denmark and Italy. This must be considered and carefully evaluated in adopting resistance management strategies to control *Lolium* spp. in cereal crops.

## Introduction

Ryegrass species (*Lolium* spp.) are obligate out-crossers with high genetic variability and fecundity (Pedersen et al., 2007; Holt et al., 2013). They are common weeds in many European countries and infest numerous cropping systems, including cereal crops, where they are considered a threat for the sustainability of cereal production. Historically, the control of *Lolium* spp. has been carried out with herbicides inhibiting acetyl coenzyme-A carboxylase (ACCase) but several populations have evolved resistance to this herbicide group. It is recognized that the diversification of the selection pressure by using herbicides with different sites of action is a key point for resistance management. Hence, the subsequent registration of acetolactate synthase (ALS) inhibitors introduced another herbicide site of action (SoA) to overcome this problem. These herbicides, able to control broad-leaved weeds as well as some grass species including *Lolium* spp., have been widely used by cereal growers. The recurrent treatments with herbicides having the same SoA have selected resistant *Lolium* spp. populations and this significantly reduces the number of herbicides available to control these weed species.

Several resistance mechanisms have been reported for *Lolium* spp. Among them, gene mutations reducing or blocking herbicide binding by conferring amino-acid changes in a target protein (Target Site Resistance, TSR) and enhanced metabolism causing accelerated herbicide degradation (one of the non-target-site resistance, NTSR mechanisms) are the main mechanisms in grass weeds such as *Lolium* spp. (Powles and Yu, 2010; Délye et al., 2013). TSR has been extensively studied in the last 20 years and many of the known mutations endowing herbicide resistance in the *ALS* (Yu and Powles, 2014) and *ACCase* (Kaundun, 2014) genes have been found in *Lolium* spp. populations (Tan et al., 2007; Scarabel et al., 2011; Han et al., 2016). Early works have established the presence of metabolic resistance to diverse herbicides such as chlorsulfuron, chlorotoluron in *Lolium rigidum* populations from Australia (Cotterman and Saari, 1992; Preston et al., 1996) and also the presence of both TSR and enhanced metabolism-based resistance (hereinafter referred as EMR) mechanisms in the same plant (Christopher et al., 1992; Tardif and Powles, 1994). From then on, EMR has been understudied and only recently, it was recognized as the predominant type of resistance to ACCase- and ALS inhibitors in grasses (Gaines et al., 2020). EMR, differently to TSR, can confer cross-resistance to herbicides with different SoA, including herbicides to which weeds have not been previously exposed (Petit et al., 2010). EMR is considered to be polygenically inherited, involving multiple genes encoding for metabolic enzymes such as cytochrome P450 monooxygenase (P450), glucosyl transferases (GT), glutathione *S*-transferases (GST), esterases and ABC transporters (Yuan et al., 2007; Duhoux and Délye, 2013; Duhoux et al., 2017). Four consistently over-expressed genes were identified in resistant individuals of *Lolium rigidum*, a close relative of *Lolium multiflorum*. These included two *P450s*, one nitronate monooxygenase (*NMO*) and one *GT* (Gaines et al., 2014, 2020). In *L. multiflorum* higher expressions of these four metabolism-related genes were reported in individuals of resistant populations from Denmark (Mahmood et al., 2016).

In Greece, the first case of *L. rigidum* resistant to both diclofop-methyl (ACCase inhibitor) and chlorsulfuron (ALS inhibitor) was reported in 2000 (Kotoula-Syka et al., 2000). Subsequently, other populations resistant only to chlorsulfuron were found and the resistance mechanism was attributed to enhanced activity of P450 in some populations and to TSR in others (Kaloumenos et al., 2012). In Italy, the first ACCase-resistant *Lolium* spp. population was recorded in the mid-1990s in central Italy (Bravin et al., 2001). Since then, ACCase-resistant cases have also spread to northern Italy and a few years ago *Lolium* spp. populations resistant to both ALS and ACCase inhibitors were recorded (Collavo et al., 2013) and they are now increasing. In Denmark, herbicide resistance in *Lolium* spp. appeared later than in Italy and Greece with the first case of *Lolium multiflorum* resistant to an ALS-inhibitor (iodosulfuron) registered in 2010 (Mathiassen, 2014). Since then, cases have been increasing and the first case of multiple-resistance to ACCase and ALS inhibitors was reported in 2010 (Heap, 2020).

*Lolium* spp. is very prone to evolve resistance and multi-resistant cases are increasing in the three countries. It was also shown that metabolic resistance evolves rapidly in *L. rigidum* when herbicides are used at low or suboptimal doses and this is an important point to consider for weed management (Neve and Powles, 2005; Manalil et al., 2011). Efforts should therefore be made to limit the evolution of resistance and to ensure the sustainability of cereal crops production. The aims of this work were (1) to determine the level of resistance to ALS and ACCase inhibitors through bioassays in twelve *Lolium* spp. populations collected in Denmark, Greece, and Italy; (2) to investigate the resistance mechanisms involved, both the detection of *ALS* and *ACCase* alleles endowing TSR and the presence of EMR mechanism. For the latter purpose, the gene expression of four herbicide metabolism related genes (*NMO*, *GST*, P450s *CYP72A1* and *CYP72A2*) was investigated through qPCR.

## Materials and Methods

### Origins of *Lolium* Populations

Seeds from *Lolium* spp. plants were collected in winter cereal fields from three European countries (Denmark, Greece, and Italy) where the control of these grass species by ALS and ACCase inhibitors was poor. After a preliminary screening of the populations, conducted in each country, 12 populations, four from each country, with a high frequency of plants resistant to both ALS and ACCase inhibitors were selected (Table 1). Additionally, susceptible reference populations from each of the three countries were included. All seed samples were cleaned and stored in paper bags at 4°C until use.

**TABLE 1:** Some details of *Lolium* spp. populations from Denmark, Greece, and Italy used in the study.

**Country**	**Location of collection**	***Lolium* species**	**Population code**	**Year of sampling**	**Selecting agents (HRAC group)**
Denmark	Randers	*L. perenne*	DK6	2017	ACCase + ALS
	Sønderborg	*L. multiflorum*	DK29	2017	ACCase + ALS
	Løgumkloster	*L. multiflorum*	DK47	2017	ACCase + ALS
	Slagelse	*L. multiflorum*	DK90	2017	ACCase + ALS
	Skive	*L. multiflorum*	DK100^a^	2016	–
	Grenå	*L. multiflorum*	DK22-M^a^	2017	–
	Haderslev	*L. perenne*	DK22-P^a^	2017	–
Greece	Arethousa	*L. rigidum*	GR9	2017	ACCase + ALS
	Drama	*L. rigidum*	GR20	2017	ACCase + ALS
	Kilkis	*L. rigidum*	GR24	2017	ACCase + ALS
	Drama	*L. rigidum*	GR30	2017	ACCase + ALS
	Leveon	*L. rigidum*	GR33^a^	2017	–
	Aliartos	*L. rigidum*	GR39^a^	2017	–
Italy	Ascoli Satriano	*L. rigidum*	IT533	2013	ACCase
	Chiarenta	*L. rigidum*	IT595	2016	ACCase + ALS
	Alessandria	*L. multiflorum*	IT609	2017	ACCase + ALS
	Caragna Piemonte	*L. multiflorum*	IT620	2017	ACCase + ALS
	Legnaro	*L. multiflorum*	IT204^a^		–

*^*a*^Reference populations.*

### Outdoor Dose-Response Experiments

Two whole-plant dose-response experiments were carried out at Legnaro (PD), Italy (45° 21′ N, 11° 58′ E). The experiments were performed as outdoor pot experiments during spring 2018 and autumn 2018 using commercial formulations of ALS and ACCase inhibitor herbicides.

To break dormancy, seeds were placed in Petri dishes on wet filter paper and vernalized at 4°C under dark conditions for 3 days. Then, seeds were placed in a germination cabinet and kept for 5 days at 25/15°C (day/night) with a 12 h photoperiod. Germinated seedlings at similar growth stages were transplanted into 16 cm diameter pots filled with a standard potting mixture (60% silty loam soil, 15% sand, 15% perlite, and 10% peat). The pots were placed outside in a semi-controlled environment and watered regularly to maintain the substrate at field capacity.

The experimental layout was a completely randomized design with three biological replicates (pots), each one with 9 seedlings. Herbicide application was carried out at BBCH 21-22 (Hess et al., 1997). All populations were treated with two ACCase inhibitors (pinoxaden and clodinafop-propargyl) and one of ALS inhibitor (mesosulfuron-methyl + iodosulfuron methyl-sodium) (Table 2). Herbicides were applied using a precision bench sprayer delivering 300 L ha^–1^ at a pressure of 215 kPa and speed of about 0.75 ms^–1^ with a boom equipped with three flat-fan (extended range) hydraulic nozzles (Teejet^®^, 11002). The herbicide doses applied ranging from 1/16 N to 4 N for the susceptible populations and from 1/8 N to 8 N for the resistant populations with N being the recommended field dose in Italy.

**TABLE 2:** Herbicides used in the dose-response experiments.

**Herbicide SoA (chemical family)**	**Commercial name (Manufacturer)**	**Active ingredient (a.i.)**	**Concentration a.i.**	**^b^Field dose (N) g a.i.ha^–1^**
ACCase (FOP^a^)	Topik 240 (Syngenta)	Clodinafop-propargyl	240 g L^–1^	60
		+ cloquintocet-mexyl (safener)	60 g L^–1^	15
ACCase (DEN^a^)	Axial pronto (Syngenta)	Pinoxaden + cloquintocet-mexyl (safener)	60 g L^–1^ 15 g L^–1^	45 10
ALS (SU^a^)	Atlantis WG (Bayer CropScience)	Mesosulfuron-methyl + iodosulfuron-methyl sodium + mefenpyr-diethyl (safener)	30 g Kg^–1^ 6 g Kg^–1^ 90 g Kg^–1^	15 3 45

*^*a*^Aryloxyphenoxypropionate (FOP); cyclohexanedione (DEN); and sulfonylurea (SU). ^*b*^Recommended Italian field dose.*

Four weeks after treatment, plant survival and shoot fresh weight were recorded for each pot and expressed as percentage of the mean of the non-treated control.

The dose-response data were analyzed using a non-linear regression analysis based on the log-logistic equation: *Y* = C + *[(D - C)/[1 + (x/I_50_)^*b*^*] where *Y* is the fresh weight or survival, *C* and *D* are the lower and upper asymptotes at very high and infinitely low doses, respectively, *b* is the slope of the curve around its inflection point, *I*_50_ is the dose giving a response equivalent to midway between the *D* and *C* parameters and *x* is the herbicide dose (g a.i.ha^–1^) (Seefeldt et al., 1995).

The ED_50_ (herbicide dose causing 50% plant mortality), GR_50_ (herbicide dose causing 50% reduction in fresh weight) and relative standard errors were estimated using GraphPad Prism 8 (GraphPad Software Inc., San Diego, CA, United States). For biological reasons and to improve the best-fit values of the parameters, the lower and upper asymptotes of plant survival and fresh weight data were constraints to 0 and 100%, respectively (Onofri, 2005). Data of each population and for each herbicide were analyzed together and an extra sum-of-squares F test, available in GraphPad Prism 8, was performed to determine if the data of the two experiments could be pooled, i.e., if one curve adequately fitted both data set.

Resistance indexes (RIs) were calculated as the ratio between the ED_50_ (or GR_50_) of the resistant and the susceptible population separately for each country. When the ED_50_ (or GR_50_) could not be determined because plant survival (or fresh weight) was higher than 50% even at the highest herbicide doses, the maximum applied dose was used as a proxy for ED_50_/GR_50_.

### Identification of Mutant *ALS* and *ACCase* Alleles

Five to ten ALS-resistant plants from the 12 *Lolium* populations were analyzed to detect the presence of mutant *ALS* and *ACCase* alleles. When no *ACCase* mutant alleles were detected another five ACCase-resistant plants were genotyped to confirm or reject the absence of mutant alleles indicating a putative non-target site resistance.

Total genomic DNA (gDNA) was extracted from 0.1 g leaf tissue using the CTAB method (Doyle and Doyle, 1987). A 1600 bp region of the CT domain of the plastidic *ACCase* gene was amplified by PCR on gDNA using the primers acclr9 and acclr6 (Table 3). The amplified region encompassed all the amino acid substitutions so far identified as conferring resistance. PCR amplifications were performed using GoTaq DNA Polymerase kit (Promega, United States) in a 25 μL mixture including 5 μL of 5 × Colorless GoTaq Flexi Buffer, dNTPs mix (0.2 mM each), MgCl_2_ (3 mM), forward and reverse primers (0.4 μM each), 0.125 μL GoTaq DNA Polymerase, and 25 ng of gDNA. The thermocycler program was as follows: 95°C for 2 min; 45 cycles of 95°C for 30 s, 57°C for 30 s, 72°C for 2 min; 72°C for 5 min. PCR products were purified with NucleoSpin Gel and PCR Clean-up kit (Macherey-Nagel GmbH & Co., Germany) following the manufacturer’s instructions. Once purified, PCR products obtained from each plant were sequenced by BMR Genomics (Padova, Italy) using primers LOL_FOR and LOL_FOR_SEQ (Table 3).

**TABLE 3:** List of primers used for the ALS and ACCase fragments amplification and sequencing.

**Primer name**	**Sequence 5′-3′**	**Target**
acclr9	ATGGTAGCCTGGATCTT GGACATG	Forward primer, *ACCase* CT domain amplification (Zhang and Powles, 2006)
acclr6	GGAAGTGTCATGCAATT CAGCAA	Forward reverse, *ACCase* CT domain amplification (Zhang and Powles, 2006)
LOL_FOR	CTGTCTGAAGAAGACTA TGGCCG	Sequencing *ACCase* gene
LOL_FOR_SEQ	GAGGTGGCTCAGCTAT GTTCCTG	Sequencing *ACCase* gene
LOL_ALS_F	CCGCAAGGGCGCCGACA TCCTCGT	Forward primer, *ALS* amplification
ALS_LOL_R	CGAAATCCTGCCATCAC CTTCCAT	Reverse primer, *ALS* amplification
ALS_LOL_FS	TCCATCACCAAGCACA ACTACCTC	Sequencing *ALS* gene

Similarly, a 1719 bp fragment of the *ALS* gene was amplified from each DNA extracted with primers LOL_ALS_F and ALS_LOL_R reported in Table 3. PCR amplifications were performed using GoTaq DNA Polymerase kit (Promega, United States) in a 25 μL total volume containing 5 μL of 5× Colorless GoTaq Flexi Buffer, 5% of DMSO (1.25 μL), dNTPs mix (0.2 mM each), MgCl_2_ (4 mM), forward and reverse primers (0.4 μM each), 0.125 μL GoTaq DNA Polymerase, and 25 ng of gDNA. The thermocycler program was as follows: 95°C for 2 min; 45 cycles of 95°C for 30 s, 60°C for 30 s, 72°C for 2 min; 72°C for 5 min. PCR products were purified as described for *ACCase* gene and sequenced by BMR Genomics using primers LOL_ALS_F and ALS_LOL_FS (Table 3).

### RNA Extraction and q-PCR

Ten populations of *Lolium* spp. were chosen for this study, four susceptible populations and six resistant populations (DK29, DK90, GR24, GR30, IT533, and IT609) that had no (or sporadic) ALS and ACCase mutated plants. Resistant plants without mutant *ALS* and *ACCase* alleles were determined as described in the previous paragraph. All populations were sown in trays placed in a glasshouse at Flakkebjerg, Denmark. At BBCH 21, twenty plants from each population were separated into two individual plants (i.e., two clones) and transplanted into pots. A week later, one clone from each plant was sprayed with mesosulfuron-methyl + iodosulfuron-methyl sodium at 30 + 6 g ha^–1^ while the other clone remained non-treated. Plant responses to herbicide treatment was visually assessed 4 weeks after application. The treated clones were rated as susceptible or resistant while the non-treated clones were cut at the soil surface and frozen in liquid nitrogen immediately after harvest for subsequent gene expression analysis.

Total RNA was extracted from 50 mg of leaf material of three individual plants of the ten populations using RNeasy Plant Mini Kit (Qiagen, Stanford, CA, United States). The quality and concentration of RNA samples were determined as reported by Mahmood et al. (2016).

The qPCR reactions were performed with the GoTaq 1-step RT-qPCR System (Promega, Madison, WI, United States) using an Applied Bioscience ViiATM7 real-time PCR system with 384 wells (Thermo Fisher Scientific, Waltham, MA, United States). Reactions were performed in triplicates and a negative control consisting of reaction mix without template was also included for each primer. Briefly, 20 μL reaction mix included 10 μL GoTaq qPCR MasterMix 2×, 4 μL RNA template, 4 μL 0.5 pmol primers (1:1 mix of forward and reverse primers), 0.4 μL GoScript RT Mix 1-Step RT-qPCR 50x, 1.6 μL nuclease-free distilled water. Reaction conditions included 50°C for 5 min followed by 10 min incubation at 95°C, then 40 cycles of 95°C for 15 s and 60°C for 1 min. One internal control gene Rab GTPase (*RGTP*) and four herbicide metabolism genes *NMO*, *GST*, *CYP72A1*, and *CYP72A2* were chosen. Primer sequences are identical to those described by Gaines et al. (2014) and available in Mahmood et al. (2016).

Threshold-cycle (C_t_) values were calculated for each reaction. Gene-specific PCR efficiency was used to calculate the expression of target genes in relation to the expression of internal reference gene. Equivalent slopes for target and internal control gene were observed in amplification plots. The ΔC_t_ value was calculated as follows: ΔC_t_ (target genes) = C_t_ (target gene) – C_t_ (reference gene), where C_t_ is the cycle number at which PCR product exceeded a set threshold. Relative transcript level (RTL) was calculated through = 1 × 2^–ΔCt^ (Pfaffl, 2006). The significance levels for each gene expression were calculated for all pairwise comparisons through a single factor ANOVA followed by Tukey HSD (Honestly Significant Difference) test.

## Results

### Dose-Response Bioassays

The extra sum-of-squares *F* test conducted on the survival and fresh weight data of each population to compare the dose-response curves obtained in the two experiments indicated that most of the curves were significantly different at *p* < 0.05. Therefore, it was not possible to estimate a common curve except for the survival data obtained with mesosulfuron + iodosulfuron (Table 4).

**TABLE 4:** Parameter estimates of the dose-response of Atlantis WG (field dose is 500 g ha^–1^ = 15 g mesosulfuron + 3 g iodosulfuron ha^–1^) on *Lolium* spp. populations.

**Population**	**Slope**	**ED_50_**	***SE***	***P*-value**	**RI**
DK6		>4000			**>50**
DK29		>3000			**>37**
DK47		>4000			**>50**
DK90		>4000			**>50**
DK100*	−2.49	55.9	4.34	0.06	
GR9	−0.71	427	75.74	0.96	**8**
GR20	−1.24	602	64.16	0.43	**11**
GR24		>4000			**>70**
GR30	−1.17	3643	690.00	0.56	**64**
GR33*	−3.17	57	3.38	0.76	
IT533	−1.83	1521	150.00	0.06	**20**
IT595		>4000			**>70**
IT609		>4000			**>70**
IT620		>4000			**>70**
IT204*	−3.17	57	3.20	0.76	

Herbicide dose that causes 50% reduction of the percentage of surviving plants (ED_50_), relative standard error (SE), curve slope, *P-*value of lack-of-fit F-test and resistance index (RI) are shown. Data for spring and autumn experiment pooled. *Susceptible populations.

For mesosulfuron + iodosulfuron the estimated ED_50_ values of the three susceptible reference populations were similar, 55.9 g ha^–1^ (=1.68 g mesosulfuron + 0.33 g iodosulfuron) for population DK100 and 57 g ha^–1^ (=1.71 g mesosulfuron + 0.34 g iodosulfuron) for populations GR33 and IT204 (Table 4). The four Danish populations exhibited high level of resistance and even at the highest herbicide dose tested (120 + 24 g a.i. ha^–1^ of mesosulfuron + iodosulfuron) plant survival was higher than 50%. Similarly, the four Italian populations were highly resistant with RIs > 70 for three populations IT595, IT609, IT620 and RI = 20 for population IT533, while the Greek populations showed RIs ranging from 8 to >70.

The reference populations showed higher ED_50_ values with clodinafop, ranging from 29 to 55 g a.i. ha^–1^, in the spring experiment than in the autumn experiment where the ED_50_ values ranged from 15 to 19 g a.i. ha^–1^. In general, the RIs of all populations were higher in the autumn experiment compared to the spring experiment, however, the ranking of populations was consistent. The four Danish populations showed low to moderate resistance to clodinafop. The RIs for plant survival were between 2.3 and 6.8 in the spring experiment and between 3.4 and 7.3 in the autumn experiment. The RIs based on fresh weight recorded slight differences between both experiments with RIs ranged between 0.9–4.8 and 0.6–8.5 in the spring and autumn experiments, respectively (Table 5).

**TABLE 5:** Dose-response experiments.

	**Clodinafop (spring experiment)**				**Clodinafop (autumn experiment)**			
**POP**	**ED_50_**	**R.I.**	**GR_50_**	**R.I.**	**ED_50_**	**R.I.**	**GR_50_**	**R.I.**
DK-6	238 (32.1)	**4.3**	206 (20.4)	**4.8**	122 (17.71)	**6.5**	2.9 (2.49)	**0.6**
DK-29	129 (15.4)	**2.3**	39 (10.2)	**0.9**	124 (16.02)	**6.6**	29.4 (7.23)	**6.6**
DK-47	147 (10.2)	**2.7**	188 (20.9)	**4.3**	63 (10.24)	**3.4**	5.4 (4.18)	**1.2**
Dk-90	372 (13.9)	**6.8**	171 (69.6)	**4.0**	136 (6.39)	**7.3**	38.1 (7.81)	**8.5**
DK-100*	55 (2.8)		43 (1.6)		18.8 (0.88)		4.5 (0.53)	
GR9	>480	**>17**	>480	**>16**	>480	**>32**	>480	**>135**
GR20	>480	**>17**	>480	**>16**	>480	**>32**	>480	**>135**
GR24	>480	**>17**	>480	**>16**	>480	**>32**	>480	**>135**
GR30	>480	**>17**	>480	**>16**	>480	**>32**	>480	**>135**
GR33*	29 (3.6)		31 (4.7)		14.8 (0.52)		3.6 (0.62)	
IT533	>480	**>11**	>480	**>16**	>480	**>27**	>480	**>104**
IT595	337 (61.1)	**8**	>480	**>16**	134 (27.93)	**8**	n.a.	
IT609	>480	**>11**	>480	**>16**	>480	**>27**	>480	**>104**
IT620	>480	**>11**	>480	**>16**	>480	**>27**	>480	**>104**
IT204*	42 (4.6)		30 (4.6)		17.5 (2.49)		4.6 (1.33)	

Clodinafop-propargyl doses (g a.i. ha^–1^) causing 50% reduction in survival (ED_50_) and fresh weight (GR_50_) on *Lolium* spp. populations and resistance indexes (RIs). Standard errors are reported in brackets. *Susceptible populations. n.a., not available because some pots were damaged.

All four Greek populations were highly resistant to clodinafop with ED_50_ and GR_50_ values that corresponded to the higher doses tested (i.e., 480 g a.i. ha^–1^) in both experiments. The resulted RIs were around 17 for both survival and fresh weight in the spring experiment and >32 or >135 in the autumn experiment. Similarly, all four Italian populations were highly resistant to clodinafop with higher values of RI recorded for the autumn experiment (Table 5).

Overall, the reference populations were completely controlled at half the recommended dose of pinoxaden (i.e., 22.5 g a.i. ha^–1^). The ED_50_ values of the three reference populations ranged between 9 and 12 g a.i ha^–1^ in both experiments. Three Danish populations (DK6, DK29, and DK47) showed a slight shift in the susceptibility to pinoxaden with RIs ranging from 1.1 to 2.6 based on plant survival and 0.9 to 3.1 based on fresh weight in both experiments while the fourth population DK90 had a higher RI. Three out of four Greek populations (GR9, GR24, GR30) were highly resistant to pinoxaden, with RIs ranging from 6 to 12.6 and from 19 to 39, based on survival in the spring and autumn experiment, respectively. The fourth population (GR20) had a lower resistance level respect to the other Greek populations (RI = 3.1 in the spring experiment and 11.4 in the autumn experiment). Finally, three of the four Italian populations (IT533, IT609, and IT620) were moderately resistant to pinoxaden, with RIs ranging between 6.3 and 7 for plant survival and between 2 and 9.2 for fresh weight. The RI values based on fresh weight were similar for the autumn experiment while the RIs based on survival were higher (Table 6).

**TABLE 6:** Dose-response experiments.

	**Pinoxaden (spring experiment)**				**Pinoxaden (autumn experiment)**			
**POP**	**ED_50_**	**R.I.**	**GR_50_**	**R.I.**	**ED_50_**	**R.I.**	**GR_50_**	**R.I.**
DK-6	15 (0.2)	**1.3**	12 (0.7)	**1.6**	13.7 (0.2)	**1.1**	9.2 (0.2)	**0.9**
DK-29	28 (1.4)	**2.3**	16 (1.6)	**2.1**	32.9 (0.9)	**2.6**	31.5(1.3)	**3.1**
DK-47	14 (0.0)	**1.2**	15 (0.4)	**2.0**	19.7 (2.7)	**1.6**	11.6 (0.9)	**1.1**
DK-90	46 (5.3)	**3.9**	15 (2.2)	**1.9**	60.1 (4.8)	**4.7**	45.0 (3.0)	**4.4**
DK-100*	12 (0.0)		8 (1.2)		12.7 (0.1)		10.1 (0.1)	
GR9	52 (8.3)	**6.0**	58 (10.8)	**6.0**	177.3 (10.4)	**19.1**	>90	**>36**
GR20	27 (1.0)	**3.1**	30 (3.3)	**3.1**	105.8 (16.3)	**11.4**	52.9 (26.5)	**21.4**
GR24	94 (15.4)	**10.8**	47 (4.8)	**4.8**	300.5 (23.2)	** 32.4**	>180	**>72**
GR30	109 (22.8)	**12.6**	73 (24.0)	**7.5**	>360	**>39**	>90	**>36**
GR33*	9 (0.0)		10 (1.8)		9.3 (0.6)		2.5 (0.2)	
IT533	85 (28.2)	**7.0**	85 (12.3)	**9.2**	211.3 (26.2)	**19.9**	36.8 (14.0)	**4.8**
IT595	20 (0.3)	**1.7**	21 (0.7)	**2.2**	18.4 (1.7)	**1.7**	25.9 (6.2)	**3.4**
IT609	84 (12.3)	**7.0**	30 (1.9)	**3.2**	68.5 (10.9)	**6.4**	51.9 (14.7)	**6.8**
IT620	77 (4.7)	**6.3**	18 (4.2)	**2.0**	130.0 (15.7)	**12.2**	24.8 (9.9)	**3.3**
IT204*	12 (0.2)		9 (1.1)		10.6 (0.0)		7.6 (0.1)	

Pinoxaden doses (g a.i. ha^–1^) causing 50% reduction in survival (ED50) and fresh weight (GR50) on *Lolium* spp. populations and resistance indexes (RIs). Standard errors are reported in brackets. *Susceptible populations.

### Mutant *ALS* and *ACCase* Alleles

Primers acclr9/acclr6 amplified a 1600 bp amplicon encompassing all codons of the *ACCase* gene known to confer resistance. Similarly, primers LOL_ALS_F/ALS_LOL_R amplified a 1719 bp amplicon encompassing all codons of the *ALS* gene conferring resistance. Both amplicons were sequenced for all the 83 plants (Table 7).

**TABLE 7:** *ALS* and *ACCase* allelic variants identified in resistant *Lolium* spp. plants from Danish, Greek, and Italian populations compared to the susceptible plant.

	***ACCase* allelic variants**						***ALS* allelic variants**					**No mutant plant/no. analyzed plants**
**Population**	**1781**	**2027**	**2041**	**2078**	**2088**	**2096**	**122**	**197**	**205**	**376**	**574**	
DK6	–	–	–	–	–	–	–	–	–	–	Leu (5)	5/5
DK29	–	–	–	–	–	–	–	–	–	–	–	0/5
DK47	–	–	–	–	–	–	–	–	–	–	Leu (4)	4/5
DK90	–	–	–	–	–	–	–	–	–	–	–	0/5
GR9	Leu (3)	–	Val (1)	–	–	–	–	Ser (3) Thr (2)	–	Asn (1)	–	5/5
GR20	–	Cys (1)	Asn (3)	Gly (1)	Arg (1)	–	–	Ser (5)	–	–	–	5/5
GR24	Leu (1)	Cys (1)	Asn (1)	Gly (1)	–	–	–	Ala (1) Gln/Leu (1)	–	–	–	5/7
GR30	Leu (3)	–	–	–	–	–	–	Ser (1)	–	–	–	4/8
IT533	Leu (2)	–	–	Gly (1)	–	Ala (1)	–	–	–	Glu (1)	–	5/10
IT595	Leu (5)	–	–	–	–	–	–	–	–	Glu (4)	Leu (4)	9/10
IT609	–	–	Asn (1)	–	–	–	–	Gln (1) Ser (1)	–	–	–	3/10
IT620	–	–	–	–	–	Ala (3)	Gly (2)	Leu (1)	Val (1)	–	–	5/8
^a^IT204	Ile	Trp	Ile	Asp	Cys	Gly	Ala	Pro	Ala	Asp	Trp	

*For each codon, the amino acid substitution identified is indicated and the number of plants carrying the mutation is reported in bracket. Dashes indicate amino acids identical to those in susceptible plant from IT-204^*a*^.*

The sequencing of ACCase amplicons revealed that all the Greek and Italian populations had plants with a mutated *ACCase* allele (Table 7). Overall, in the Greek populations, six different *ACCase* mutant alleles were detected: Leu_1781_, Cys_2027_, Asn_2041_, Val_2041_, Gly_2078_, and Arg_2088_. In population GR30 only one type of mutant *ACCase* allele was detected (Leu_1781_), in population GR9 two types (Leu_1781_ and Val_2041_), in population GR20 four types (Cys_2027_, Asn_2041_, Gly_2078_, and Arg_2088_) as in population GR24 (Leu_1781_, Cys_2027_, Asn_2041_, and Gly_2078_). In addition, different mutant *ACCase* alleles were found in the same plant of population GR20.

Four different types of *ACCase* mutant alleles were found in plants from Italy. Populations IT595, IT609 and IT620 showed only one *ACCase* mutant allele each – Leu_1781_, Asn_2041_ and Ala_2096_, respectively. In population IT533, three mutant alleles were identified: Leu_1781_, Gly_2078_, and Ala_2096_. In contrast, no mutant *ACCase* alleles were identified in any of the 20 plants analyzed from the Danish populations. It is noteworthy that several plants with no mutated *ACCase* allele were also identified within two Greek populations (GR24 and GR30) and in all the Italian populations (Table 7).

Six types of mutant *ALS* alleles (Ala_197_, Gln_197_, Leu_197_, Ser_197_, Thr_197_, and Asn_376_) were identified in the plants of the Greek populations. In populations GR20 and GR30 only one type of *ALS* mutant allele was detected (Ser_197_), while in populations GR9 three types (Ser_197_, Thr_197_, and Asn_376_) and in GR24 three types (Ala_197_, Gln_197_, and Leu_197_) were identified. In three of the four Italian populations more than one mutant *ALS* allele was detected: Glu_376_ and Leu_574_ in IT595, Gln_197_ and Ser_197_ in IT609 and Gly_122_, Leu_197_ and Val_205_ in IT620. The fourth population, IT533, had only the Glu_376_ allelic variant. Conversely, the plants of the Danish populations showed only one type of mutant *ALS* allele (Leu_574_), and only in two populations (DK6 and DK47) (Table 7). The ALS and ACCase sequences presented can be found in a specific repository (Panozzo and Scarabel, 2020).

### Gene Expression of Herbicide Metabolism Related Genes

Six of the resistant populations (DK29, DK90, GR24, GR30, IT533, and IT609) that had plants with no *ALS* and *ACCase* mutant alleles were further studied to determine the expression patterns of four genes, *NMO*, *GST*, *CYP72A1*, *CYP72A2* known to be involved in herbicide metabolism. Four susceptible *Lolium* spp. populations were also examined (DK39, DK100, DK22M and DK22P).

**FIGURE 1:**
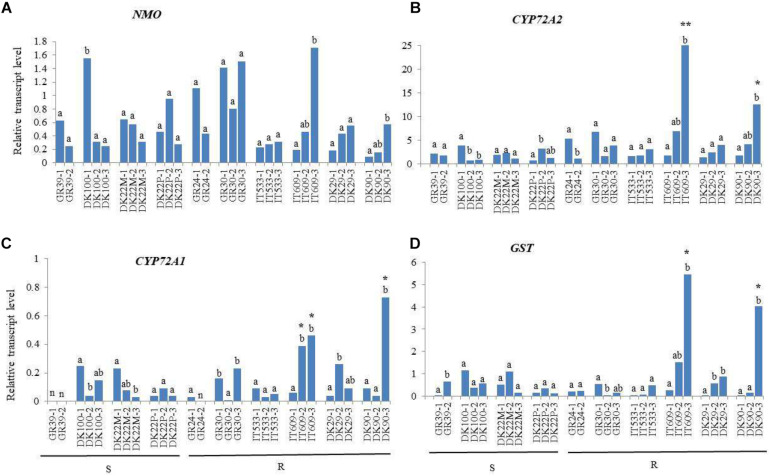
Relative transcript levels of *NMO* **(A)**, *CYP72A2* **(B)**, *CYP72A1* **(C)**, and *GST* **(D)** in leaves of *Lolium* spp. plants resistant (R) or susceptible (S) to mesosulfuron-methyl + iodosulfuron-methyl relative to the internal reference gene *RGTP.* Significant differences between plants of the same population are indicated with different letters. Asterisks denote significant differences between R plant compared to all S plants tested (**) or compared to plants of only one S population (*). *n* = samples with Ct values below no template control.

For all four genes, significant differences between plants of the same population were observed. For example, the expression of *NMO* in plant DK100-1 was around 5.5 fold higher than in plants 2 and 3 (Figure 1A) and the expression in plant IT609-3 was 9 and 3.7 fold higher respect to plant 1 and 2, respectively. These differences were observed within all populations, except population IT533, implying that in general *Lolium* populations are heterogeneous for the gene expression of one or more herbicide metabolism genes. No significant differences in the expression of NMO were observed between susceptible and resistant populations (Figure 1A).

Among the genes studied, *CYP72A2* was expressed at the highest level in the resistant plant (IT609-3) with a relative expression value of 24.99 while the lowest expression was found in the susceptible plant DK22P-1 with RTL value of 0.72. The expression of *CYP72A2* was significantly higher in plant IT609-3 compared to all the susceptible plants analyzed, and in the resistant plant DK90-3 compared to plants from the susceptible population DK22M, from 5 to 11-fold up-regulated. Other resistant plants, GR24-1, GR30-1 and IT609-2 exhibited higher expression respect to few susceptible plants. For example, *CYP72A2* was 9.4 fold higher expressed in plant GR30-1 respect to the susceptible plant DK22P-1 but only 1.8 fold higher respect to plant DK100-1 (Figure 1B).

The *CYP72A1* gene was expressed at a relatively low level as indicated by its generally low RTL values ranging from 0.03 to 0.73 However, a significant (*p* < 0.001) increased expression was found in plants IT609-2, IT609-3 and DK90-3. *CYP72A1* was 8-fold up-regulated in the resistant plant IT609-3 compared on average to the susceptible plants of population DK22P and 7-fold up-regulated in the resistant plant IT609-2 compared to the same susceptible population. Plant DK90-3 exhibited the highest relative expression value for *CYP72A1* (0.73) and the gene was 13-fold up-regulated compared on average to the susceptible plants of population DK22P and was also significantly up-regulated respect to plants DK100-2, DK100-3, DK22M-2 and DK22M-3 with 18, 5, 9 and 24-fold up-regulation, respectively (Figure 1C).

The *GST* gene showed significantly (*p* < 0.001) up-regulation in plant IT609-3 and DK90-3, whereas among the susceptible plants, population DK22P showed very low relative expression (on average RTL value of 0.19) as well as plant GR39-1 (RTL = 0.04) and DK22M-3 (RTL = 0.15). *GST* was 30-fold up-regulated in the resistant plant IT609-3 compared on average to the susceptible plants of population DK22P and was also significantly up-regulated compared to some plants of other susceptible populations, GR39-1, DK22M-1, DK22-M3 and DK100-2 with 136, 11, 36 and 14-fold up-regulation, respectively. Similarly, *GST* was 22-fold up-regulated in plant DK90-3 in comparison with the susceptible plants of population DK22P and was also significantly up-regulated compared to some plants of other susceptible populations, GR39-1, DK22-M3 and DK100-2 with 101, 27 and 11-fold up-regulation, respectively (Figure 1D). *GST* showed the highest differences in expression between susceptible and resistant populations respect to the other genes studied.

## Discussion

### Occurrence of Multi-Resistant *Lolium* spp.

This study confirmed the occurrence of *Lolium* spp. populations multi-resistant to ALS and ACCase inhibitors in Denmark, Italy and Greece and highlighted differences in the pattern and level of resistance among countries and populations. TSR appears to be responsible for the resistance status of Greek populations, and for most of the Italians. Conversely, resistance in Danish populations is totally endowed by NTSR mechanism in case of ACCase inhibitors and by both NTSR and TSR in case of ALS inhibitors.

While all populations were highly resistant to mesosulfuron-methyl + iodosulfuron-methyl, the susceptibility to both ACCase inhibitors, clodinafop-propargyl and pinoxaden was generally much lower in the Danish populations compared to the Italian and Greek ones. A low resistance level has often been associated to the presence of non-target-site resistance mechanisms, however, in some cases the level of resistance can be higher due to the build-up over time of different NTSR mechanisms (Cocker et al., 2001; Kaundun, 2014). Among the investigated populations, the level of resistance to pinoxaden varied among populations in each country and among countries. These differences in the pattern and level of resistance could be related to different cropping practices and herbicides used in the three countries (Llewellyn and Powles, 2001; Owen et al., 2014). The herbicide pinoxaden is not authorized in Denmark while it is frequently used as a post-emergence application in Italian and Greek winter cereals fields. This supports the higher resistance indexes generally observed in the Italian and Greek populations. However, even if pinoxaden is not used, a low resistance level to pinoxaden has been detected in one Danish population. Instead, a low but clear resistance to clodinafop was found. This type of resistant phenotype as well as the absence of mutated *ACCase* alleles strongly suggests the presence of a NTSR mechanism.

### Diversity of *ACCase* and *ALS* Alleles Endowing Resistance

*ACCase* variant alleles endowing resistance were present in all the Greek and Italian populations studied. Overall, six different types of *ACCase* variant alleles were detected in the Greek populations: Leu_1781_, Cys_2027_, Asn_2041_, Val_2041_, Gly_2078_, and Arg_2088_ and four in the Italian populations: Leu_1781,_ Asn_2041_, Gly_2078_, and Ala_2096_. Depending on the population considered, one to four different *ACCase* alleles were observed in the same population. Similar results were reported by Yu et al. (2007) who found different *ACCase* mutant alleles in Italian *Lolium* populations resistant to clethodim and showed that homozygous plants having Leu_1781_, Gly_2078_ or Arg_2088_ were also resistant to other ACCase inhibitors including clodinafop and pinoxaden. In a subsequent study conducted on Italian *Lolium* spp. populations resistant to pinoxaden, the same *ACCase* variant alleles as detected in our work were found, except for Cys_2027_ (Scarabel et al., 2011). In contrast, in all the four Danish populations investigated no ACCase variant alleles were detected indicating that target site resistance is not present.

The analyses of the *ALS* gene indicated that only one *ALS* variant allele (Leu_574_) endowing resistance to mesosulfuron-methyl + iodosulfuron-methyl was present in two Danish populations while in the other two, no *ALS* variants were detected. Conversely, in the Greek and Italian populations different *ALS* variant alleles were found, six (Ala_197_, Gln_197_, Leu_197_, Ser_197_, Thr_197,_ Asn_376_) in the Greek populations and seven in the Italian ones (Gly_122,_ Gln_197,_ Leu_197,_ Ser_197_, Val_205_, Glu_376_, Leu_574_) and diversity of *ALS* alleles was detected in some populations. The Italian populations showed amino acid substitutions at five different codons of the *ALS* gene. This is in accordance with the study of Yu et al. (2008), who, in a single Australian population, identified six different mutations in the *ALS* gene endowing resistance to chlorsulfuron. This is not surprising as *Lolium* spp. are obligate cross-pollinated species and therefore pollination among resistant plants from neighboring fields can occur within 3 km distance increasing the genetic heterogeneity of the *Lolium* plants (Busi et al., 2008). In the Greek populations, the majority of amino acid substitutions endowing resistance was observed at codon Pro-197 and this is in accordance with previous findings (Kaloumenos et al., 2012; Anthimidou et al., 2020). This substitution was frequently reported in numerous grass weeds and it usually confers resistance only to sulfonylureas (such as mesosulfuron-methyl + iodosulfuron-methyl) (Yu and Powles, 2014). Instead, the Leu_574_ *ALS* allele, present only in two Danish populations, endows high resistance to all chemical group of ALS inhibitors (Heap, 2020). The variability in the ALS mutations detected in the three countries confirms the differences observed in the cross-resistance pattern.

Some plants in two Greek populations (GR24 and GR30) and in all the Italian populations showed no amino acid substitutions endowing resistance to ALS and ACCase. This suggests that a different resistance mechanism (i.e., NTS) is likely present.

### Metabolism-Based Resistance in *Lolium* spp.

Enhanced metabolism-based resistance is considered the prevalent resistance mechanism in grass weeds and its complex genetic control (polygenic control) involves the regulation of specific genes (Délye, 2013). The cytochromes P450 belong to a supergene family and are involved in all the pathways of plant secondary metabolism (Werck-Reichhart and Feyereisen, 2000). They play a major role in the phase I of metabolic herbicide detoxification and in the coordination with the GST enzymes involved in phase II of herbicide detoxification (Cocker et al., 2001; Yuan et al., 2007). GSTs include a large, complex gene family in plants that catalyze the conjugation to various substrates and oxidatively produced compounds to reduced glutathione, which facilitates their metabolism and sequestration (Dalton et al., 2009). Nitronate monooxygenase is a flavin-dependent enzyme that oxidizes anionic alkyl nitronates. It is active on a broad range of substrates containing primary and secondary nitro groups and its involvement in detoxification of propionate-3-nitronate was reported in yeast and bacteria (Gadda and Francis, 2010). In *A. thaliana*, NMO was found to be associated with detoxification of the allelochemical benzoxazolin (Baerson et al., 2005).

The high expression of *CYPs* and *GST* is expected to enhance herbicide degradation in resistant plants (Délye, 2013). The expression level of both *CYPs* studied (*CYP72A2* and *CYP72A1*) varied broadly from plant to plant and the same was observed for the gene *GST*. This variability of expression between plants implies that the populations are generally heterogeneous for the gene expression of one or more herbicide metabolism genes. Similar findings were reported by Duhoux and Délye (2013) who reported that the expression of five *CYP* genes, both constitutive and herbicide-induced, varied broadly from plant to plant in a French *Lolium* spp. population. Despite the variability among plants, our data showed that the expression levels of *CYP72A1*, *CYP72A2*, and *GST* were significantly higher in resistant plants in population IT609 and DK90 compared to the expression level of the susceptible plants. This proves that an enhanced herbicide degradation is present in these plants and highlights the evolution of metabolic based resistance in these *Lolium* populations. *CYP72A* gene was found to be involved in metabolic resistance to diclofop in *L. rigidum* (Gaines et al., 2014). Moreover, enhanced *GST* expression was shown to determine an acceleration in the herbicide degradation in a clodinafop-resistant *L. rigidum* population (Gaines et al., 2014) and in Danish *L. multiflorum* population (Mahmood et al., 2016).

No clear distinction in *NMO* expression was observed between resistant and susceptible plants. This gene was identified as candidate gene by RNAseq transcriptome analysis involved in metabolism-based diclofop resistance in *L. rigidum* (Gaines et al., 2014). *NMO* was found to be two-fold up-regulated in glufosinate-resistant *Amaranthus palmeri* respect to susceptible plants, both constitutively and herbicide-induced (Salas-Perez et al., 2018).

The no clear distinction of expression in the genes studied that are specific to the resistant plant may be related to the fact that additional genes not studied here are involved in the herbicide metabolization. Two cytochrome P450 genes (*CYP81A12* and *CYP81A21*) were found to be overexpressed and associated with resistance to ALS inhibitors in *Echinochloa phyllopogon* (Iwakami et al., 2014). Recently, these genes were found to be involved in the detoxification of ACCase inhibitors in multiple herbicide resistant *E. phyllopogon* (Iwakami et al., 2019). Because of the high number and variability of CYPs and GSTs, plants have the potential to overcome the herbicide treatment trough a concerted action of several genes. It was demonstrated that the genetic control of P450 metabolism-based resistance mechanism in *Lolium rigidum* is governed by a set of genes that varied among plants, even in a given population (Busi et al., 2011).

In conclusion, in the present plant material, IT609-3 and DK90-3 exhibited high expression of *GST, CYP72A1*, and *CYP72A2* genes constitutively, implying that herbicide resistance for these populations could be attributed to an elevated level of herbicide metabolism.

### Coevolution of Resistance Mechanisms

In the last decade there has been an increase in multi-resistant *Lolium* spp. populations in Europe (Heap, 2020). This work highlights the presence of a wide variety of multi-resistant *Lolium* spp. populations in Denmark, Greece, and Italy and provides strong evidence that two different resistance mechanisms (TSR and NTSR metabolism-based resistance) co-evolved.

A diversity of mutant *ALS* and *ACCase* alleles were detected among plants of the Greek and Italian populations indicating a high population heterogeneity. In the Danish populations, only one type of mutant *ALS* allele was found. However, in both situations, evolution of target-site resistance is suggested to be the result of a strong selection pressure imposed by the herbicides used. The frequent use of the ALS inhibitor, mesosulfuron + iodosulfuron and lower use of ACCase inhibitors in Danish cereals fields compared with the common herbicide usages in Italy and Greece may explain this difference.

The high variability among plants observed in the expression profile of the four genes involved in the metabolism of mesosulfuron + iodosulfuron indicates that even if plants are subjected to the same herbicide selective pressure, a different weed response to the chemical agent can occur. It is likely that since EMR is a polygenic trait, the evolution process over time allows accumulation of various favorable traits able to increase the survival of the plant and the transmission to the next generation.

The presence of different resistance mechanisms increases the complexity of resistant *Lolium* spp. management in cereal fields. From a practical point of view, TSR determines resistance to herbicides with the same SoA, while metabolism-based resistance can not only endow resistance to the selecting herbicides but also confers cross-resistance to herbicides having different SoA. Numerous genes can be involved in metabolism-based resistance and, according to their regulation, they can confer resistance to different chemicals (Gaines et al., 2014). Therefore, EMR mechanism observed in some *Lolium* populations investigated in this study is of concern because the simple rotation over the years of ALS and ACCase inhibiting herbicides will not be effective, as pointed out by Collavo et al. (2013).

## Conclusion

This work displays the heterogeneity in the pattern and level of resistance to ALS and ACCase inhibitors in Danish, Greek, and Italian *Lolium* spp. populations and demonstrates the presence of target-site resistance and coexistence of metabolic based herbicide resistance mechanism in populations from Denmark and Italy. The potential of evolution of enhanced metabolism-based resistance is an important threat to consider for improving practices against resistance.

## Data Availability Statement

The raw data supporting the conclusions of this article will be made available by the authors, without undue reservation.

## Author Contributions

All authors contributed to the design of this study, shared plant materials, contributed to give comments, revised the manuscript, and read and approved the final manuscript. DL, LS, MK, SM, and SP performed the experiments and analyzed the data. LS wrote the first draft of the manuscript.

## Conflict of Interest

The authors declare that the research was conducted in the absence of any commercial or financial relationships that could be construed as a potential conflict of interest.
