# Supplementary material for: Diversified Resistance Mechanisms in Multi-Resistant Lolium spp. in Three European Countries
Source: Front Plant Sci. 2020 Dec 15;11:608845. doi: 10.3389/fpls.2020.608845 (PMC7769757; doi:10.3389/fpls.2020.608845)
Supplement: Supplementary file 1 [file Table_1.DOCX]

Table S1. Expression of herbicide metabolism genes in *Lolium* spp. populations: relative transcript level (RTL), ΔC_t_ and standard S.E. were based on reference to internal reference gene *RGTP*.

The ΔCt ± STD used for statistical analysis are shown in parenthesis. The significance levels for each gene were calculated for all pairwise comparisons by an initial analysis of variance by a single factor Anova followed by Tukey HSD (Honestly Significant Difference) test.

The nd indicates samples with Ct values below “no template” control.

One asterisk (*) Indicates where one plant is significant different (p<0.0010) to a plant from the same population. Two asterisks (**) indicates where one plant is significant different (p<0.0010) to two plants from the same population.

|  | *GST* | *CYP72A1* | *CYP72A2* | *NMO* |
| --- | --- | --- | --- | --- |
| Plant | RTL (ΔC_t_ ± S.E) | RTL (ΔC_t_ ± S.E.) | RTL (ΔC_t_ ± S.E.) | RTL (ΔC_t_ ± S.E.) |
|  |  |  |  |  |
| GR39-1 | 0.04 (4.79±0.31) | nd | 2.14 (-3.29±0.37) | 0.63 (0.66±0.31) |
| GR39-2 | 0.66 (0.60±0.57)* | nd | 1.82 (-2.58±0.57) | 0.25 (2.01±0.78) |
| GR39-3 | 0.02 | 0.06 | 0.33 | 0.08 |
|  |  |  |  |  |
| DK100-1 | 1.15 (-0.21±0.27) | 0.25 (2.00±0.24)* | 3.85 (-5.83±0.28)** | 1.55 (-0.63±0.29)** |
| DK100-2 | 0.38 (1.41±0.84) | 0.04 (4.56±0.85) | 0.77 (1.15±0.85) | 0.31 (1.67±0.89) |
| DK100-3 | 0.58 (0.80±0.10) | 0.15 (2.77±0.10) | 0.93 (0.33±0.11) | 0.25 (2.00±0.09) |
|  |  |  |  |  |
| DK22M-1 | 0.50 (0.99±0.72) | 0.23 (2.09±0.66)* | 1.93 (-2.85±0.68) | 0.65 (0.63±0.68) |
| DK22M-2 | 1.09 (-0.12±0.42) | 0.08 (3.56±0.45) | 2.38 (-3.75±0.40) | 0.57 (0.81±0.29) |
| DK22M-3 | 0.15 (2.75±0.03) | 0.03 (5.30±0.08) | 1.12 (-0.48±0.03) | 0.31 (1.69±0.03) |
|  |  |  |  |  |
| DK22P-1 | 0.14 (2.85±0.06) | 0.04 (4.76±0.26) | 0.72 (1.44±0.10) | 0.46 (1.12±0.08) |
| DK22P-2 | 0.33 (1.58±0.24) | 0.09 (3.42±0.24) | 3.21 (-5.04±0.13)* | 0.95 (0.08±0.08) |
| DK22P-3 | 0.11 (3.17±0.08) | 0.04 (4.72±0.24) | 1.27 (-1.02±0.12) | 0.28 (1.86±0.06) |
|  |  |  |  |  |
| GR24-1 | 0.20 (2.32±0.03) | 0.03 (5.28±0.09) | 5.30 (-7.22±0.05)* | 1.11 (-0.15±0.08) |
| GR24-2 | 0.23 (2.14±0.07) | nd | 1.17 (-0.67±0.12) | 0.43 (1.23±0.25) |
|  |  |  |  |  |
| GR30-1 | 0.55 (0.85±0.19)* | 0.16 (2.69±0.19) | 6.77 (-8.27±0.13) | 1.41(-0.49±0.01) |
| GR30-2 | 0.05 (4.28±0.09) | 0.01 (6.24±0.14)** | 1.70 (-2.29±0.06) | 0.80 (0.32±0.05) |
| GR30-3 | 0.15 (2.77±0.05) | 0.23 (2.15±0.26) | 3.91 (-5.90±0.07) | 1.51 (-0.59±0.03) |
|  |  |  |  |  |
| IT533-1 | 0.05 (4.21±0.03) | 0.09 (3.42±0.02) | 1.64 (-2.15±0.03) | 0.23 (2.15±0.07) |
| IT533-2 | 0.06 (4.00±0.03) | 0.03 (5.22±0.02) | 1.77 (-2.46±0.10) | 0.28 (1.82±0.01) |
| IT533-3 | 0.48 (1.05±0.08) | 0.05 (4.29±0.12) | 3.10 (-4.89±0.29) | 0.31 (1.69±0.29) |
|  |  |  |  |  |
| IT609-1 | 0.25 (2.02±0.02) | 0.06 (4.07±0.02)** | 1.73 (-2.36±0.07) | 0.19 (2.42±0.03) |
| IT609-2 | 1.53 (-0.61±0.01) | 0.39 (1.37±0.09) | 6.87 (-8.34±0.12) | 0.46 (1.13±0.03) |
| IT609-3 | 5.46 (-2.45±0.03)* | 0.46 (1.13±0.12) | 24.99 (-13.93±0.09)* | 1.71 (-0.77±0.02)* |
|  |  |  |  |  |
| DK29-1 | 0.01 (6.69±0.10)** | 0.04 (4.73±0.04) | 1.38 (-1.38±0.03) | 0.18 (2.50±0.02) |
| DK29-2 | 0.58 (0.78±0.02) | 0.26 (1.96±0.00)* | 2.44 (-3.87±0.05) | 0.43 (1.23±0.03) |
| DK29-3 | 0.88 (0.18±0.40) | 0.09 (3.40±0.55) | 4.03 (-6.03±0.48) | 0.55 (0.86±0.55) |
|  |  |  |  |  |
| DK90-1 | 0.03 (5.28±1.89) | 0.09 (3.53±0.10) | 1.77 (-2.47±0.02) | 0.09 (3.51±0.11) |
| DK90-2 | 0.14 (2.80±0.58) | 0.04 (4.82±0.15) | 4.17 (-6.18±0.06) | 0.16 (2.66±0.05) |
| DK90-3 | 4.04 (-2.02±0.14)** | 0.73 (0.45±0.17)** | 12.50 (-10.93±0.04)* | 0.57 (0.80±0.01)* |
